# Supplementary material for: Dual roles of microbes in mediating soil carbon dynamics in response to warming
Source: Nat Commun. 2024 Jul 31;15:6439. doi: 10.1038/s41467-024-50800-4 (PMC11291496; doi:10.1038/s41467-024-50800-4)
Supplement: Supplementary file 1 — Supplementary information [file 41467_2024_50800_MOESM1_ESM.pdf]

Supplementary Information for

Dual roles of microbes in mediating soil carbon dynamics in response to warming

Shuqi Qin[1,2], Dianye Zhang[1,2], Bin Wei[1,2,3], and Yuanhe Yang[1,2,3*]

[1]State Key Laboratory of Vegetation and Environmental Change, Institute of Botany, Chinese Academy of Sciences, Beijing, China.
[2]China National Botanical Garden, Beijing, China.
[3]University of Chinese Academy of Sciences, Beijing, China.

[*]**Corresponding author:** Dr. Yuanhe Yang, tel.: +86-10-6283 6638, E-mail: yhyang@ibcas.ac.cn

Supplementary Materials

Supplementary Notes 1-2

Supplementary Figures 1-10

Supplementary Tables 1-5

Supplementary Note 1 Microbial community composition under the control and warming treatments

Topsoil prokaryotic communities were predominantly composed of members of the phyla *Proteobacteria*, *Acidobacteriota*, *Verrucomicrobiota*, and *Bacteroidota* under both the control and warming treatments, accounting for more than 70% of the relative abundance of all the phyla (Supplementary Fig. 2a). Differentially abundant prokaryotic taxa between the two treatments were then identified by linear discriminant analysis (LDA) effect size (LEfSe) analysis. The results revealed that at the phylum and class level, no taxa showed significant difference in relative abundance between warming and control conditions. At the order level, *Phycisphaerales*, *Holophagales*, and *Actinomarinales* were more abundant in the control plots. At the family level, *env.OPS_17*, *Omnitrophales*, and *Holophagaceae* exhibited enrichment under control, while *Methyloligellaceae* and *Chromobacteriaceae* had higher relative abundance under warming. In addition, genera *env.OPS_17*, *Edaphobaculum*, *Omnitrophales*, *966-1*, and *Geothrix* were more abundant under control condition, and *Burkholderia-Caballeronia-Paraburkholderia* showed enrichment under warming (Supplementary Fig. 2b).

Topsoil fungal communities were primarily comprised of members of the phyla *Ascomycota*, *Basidiomycota* and *Mortierellomycota* under control and warming conditions, making up more than 90% of the relative abundance of all the phyla (Supplementary Fig. 2c). LEfSe analysis further identified differentially abundant

fungal taxa from phylum to genus level (Supplementary Fig. 2d). Specifically, the relative abundance of phylum *Zoopagomycota*, classes *Lecanoromycetes* and *Zoopagomycetes*, and orders *Chaetothyriales* and *Zoopagales* was much higher under warming. At the family level, *Massarinaceae* exhibited enrichment for control plots, while *Stachybotryaceae* and *Calloriaceae* were more abundant under warming. At the genus level, *Orbilia*, *Petrakia* and *Elasticomyces* showed higher relative abundance under control condition, whereas *Sphaerellopsis*, *Lapidomyces* and *Acremonium* revealed enrichment under the warming treatment.

Supplementary Note 2 Network descriptions, keystone node identification and module-eigengene analysis

Network descriptions. The empirical prokaryotic and fungal networks were significantly different from the corresponding random networks (Supplementary Table 4), and all showed general features of complex networks including scale free, small world, and modular[1,2,3]. Briefly, all the empirical networks had a power-law degree distribution (R^2 from 0.92 to 0.998), indicative of scale-free property. The average path distances ranged from 2.94 to 4.97, which were comparable to those in other networks exhibiting small-world property[2]. In addition, the empirical networks had higher modularity values than those of the corresponding random networks, suggesting that these networks appeared to be modular. To better compare the modular structure of networks between warming and control conditions, relative modularity (*RM*) which could reflect how modular a network is as compared with the modularity of the random networks was calculated as follows[4]:

$$RM = \frac{M - \overline{M_r}}{\overline{M_r}} \quad (1)$$

where M is the modularity of the empirical network, and $\overline{M_r}$ is the mean value of modularity of the random networks. The results revealed that experimental warming enhanced the relative modularity of both prokaryotic and fungal networks (Supplementary Fig. 3a, c), suggesting altered network structure under warming condition.

Keystone node identification. To identify keystone nodes, we firstly classified the

topological role of each node in the network according to its within-module connectivity (Z_i) and among-module connectivity (P_i)[5,6]. Z_i reflects how well node *i* is connected to other nodes within its own module, while P_i describes the degree to which node *i* is connected to different modules. Based on each node's position in the *ZP*-parameter space, we adopted the simplified classification[5] to identify the topological role of each node as follows: (i) Connectors ($Zi \leq 2.5$, $Pi > 0.62$) which "glue" several modules together, (ii) Module hubs ($Zi > 2.5$, $Pi \leq 0.62$) which are highly connected to many nodes within their own modules, (iii) Network hubs ($Zi > 2.5$, $Pi > 0.62$) which are important to the coherence of both its own module and the network, and (iv) peripherals ($Zi \leq 2.5$, $Pi \leq 0.62$) that have a few links within its own module and rarely any to the nodes in other modules. Connectors, module hubs, and network hubs were then referred to as keystone nodes as done by previous studies[4,7]. According to the criteria, no keystone node was detected in the prokaryotic and fungal networks under control (Supplementary Fig. 3b, d). 6 nodes (2% of total nodes) were identified as keystone nodes in the prokaryotic network under warming, including 4 connectors and 2 module hubs (Supplementary Fig. 3b). For the fungal network under warming, 1 connector and 1 module hub were identified as keystone nodes (2% of total nodes; Supplementary Fig. 3d). These results indicated that experimental warming would alter network structure at the keystone node level[4].

Module-eigengene analysis. We performed module-eigengene analysis to explore how network modules were connected to soil and plant variables under control and warming

conditions, respectively. Specifically, singular value decomposition analysis was firstly used to define module eigengene which could represent the variation in relative abundance of amplicon sequence variants (ASVs) within a module[2,8] (modules with more than 5 nodes were considered in this analysis). Most of the eigengenes (23/29) could explain more than 60% of the variations in relative ASV abundance across different samples. Then, we calculated correlations of these eigengenes with soil and plant variables. The results revealed that in prokaryotic network under control, modules #1 and #2 were negatively correlated with soil pH, while modules #4, #6 and #8 were positively associated with root biomass, soil pH, and Normalized Difference Vegetation Index (NDVI), respectively (Supplementary Fig. 4c). In prokaryotic network under warming, modules #2, #7, and #9 were positively correlated with soil temperature, and modules #2 and #7 were also positively correlated with soil pH. In contrast, modules #1, #3, and #9 were negatively correlated with the content of NO_3^--N, and module #1 was negatively associated with NDVI (Supplementary Fig. 4e). In addition, positive correlations were detected between module #1 and soil pH, and module #4 and NDVI, while negative associations were found between module #3 and root biomass, and module #6 and the content of NO_3^--N in fungal network under control condition (Supplementary Fig. 4d). Fungal modules #1 and #4 were negatively correlated with NDVI under warming (Supplementary Fig. 4f). These results indicated that experimental warming would alter the associations of network modules with environmental variables.

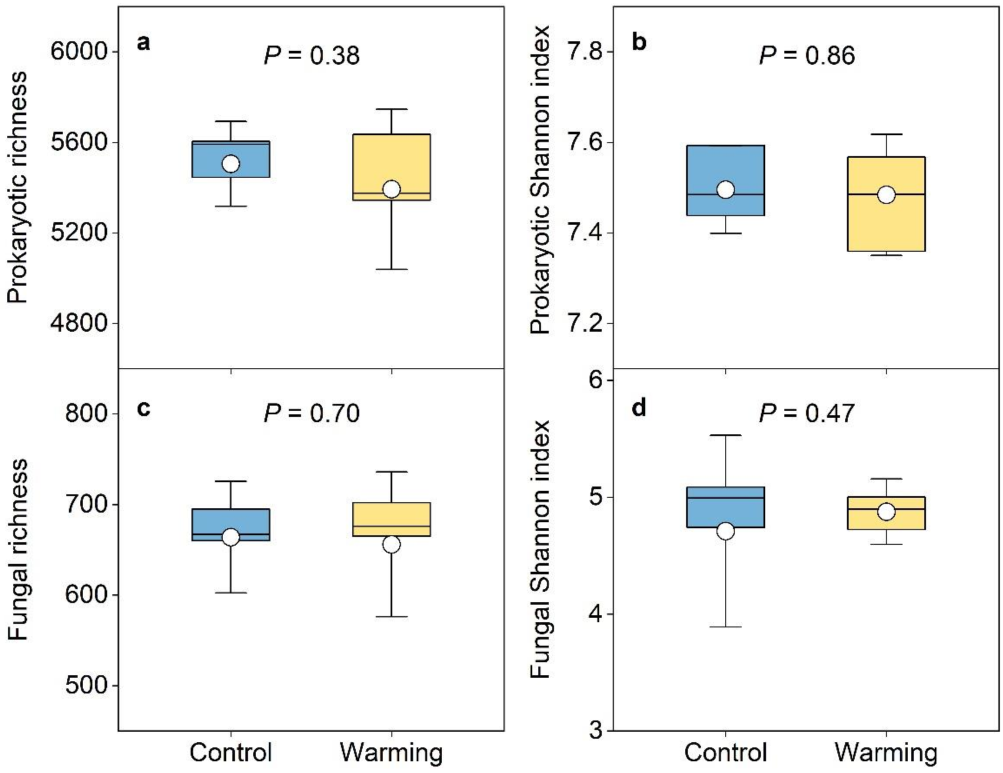

Supplementary Fig. 1 | Warming effects on topsoil microbial alpha diversity. a, b, Comparison of richness (a) and Shannon-Wiener index (b) of prokaryotes. **c, d,** Comparison of richness (c) and Shannon-Wiener index (d) of fungi. Box represents the interquartile range, with blue and yellow indicating control and warming, respectively. Horizontal line and circle within the box show the median and mean value, respectively. The whisker denotes SD (n = 10, independent samples). Paired samples t-tests (two-sided) were used to determine warming effects on means of alpha diversity. Source data are provided as a Source Data file.

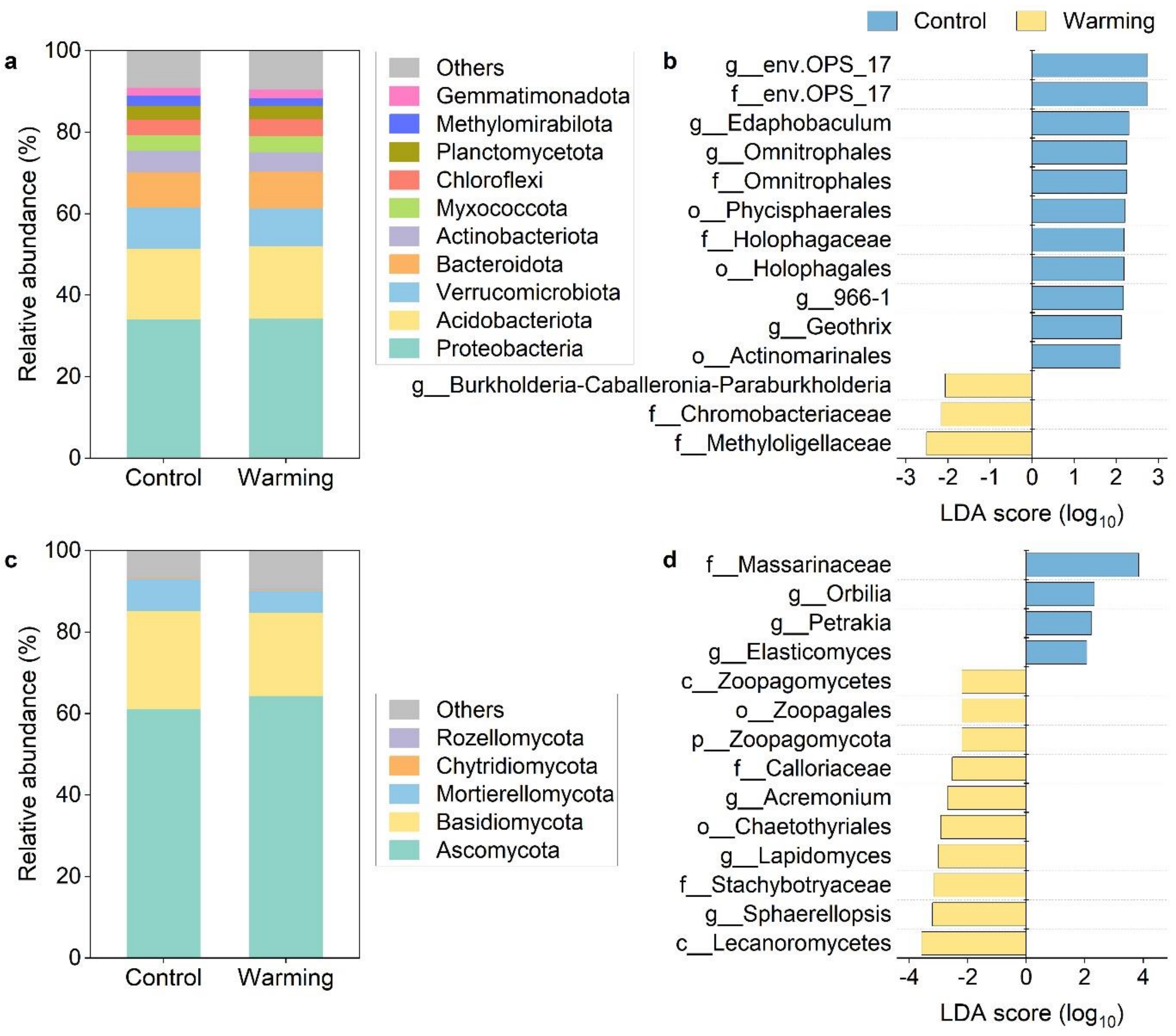

a
b
c
d
Relative abundance (%)
Control
Warming
Others
Gemmatimonadota
Methylomirabilota
Planctomycetota
Chloroflexi
Myxococcota
Actinobacteriota
Bacteroidota
Verrucomicrobiota
Acidobacteriota
Proteobacteria
g__env.OPS_17
f__env.OPS_17
g__Edaphobaculum
g__Omnitrophales
f__Omnitrophales
o__Phycisphaerales
f__Holophagaceae
o__Holophagales
g__966-1
g__Geothrix
o__Actinomarinales
g__Burkholderia-Caballeronia-Paraburkholderia
f__Chromobacteriaceae
f__Methyloligellaceae
LDA score ($\log_{10}$)
Rozellomycota
Chytridiomycota
Mortierellomycota
Basidiomycota
Ascomycota
f__Massarinaceae
g__Orbilia
g__Petrakia
g__Elasticomyces
c__Zoopagomycetes
o__Zoopagales
p__Zoopagomycota
f__Calloriaceae
g__Acremonium
o__Chaetothyriales
g__Lapidomyces
f__Stachybotryaceae
g__Sphaerellopsis
c__Lecanoromycetes

Supplementary Fig. 2 | Responses of topsoil microbial community composition to experimental warming. a, **c**, Prokaryotic (a) and fungal (c) community composition at phylum level. **b, d,** Histogram of the linear discriminant analysis (LDA) scores computed for prokaryotic (b) and fungal (d) taxa differentially abundant (LDA score > 2, unadjusted $P < 0.05$) between the warming and control treatments. Taxa enriched under control condition are indicated by a positive LDA score (blue), and taxa enriched under warming are indicated by a negative score (yellow). p_, phylum; c_, class; o_, order; f_, family; g_, genus. Source data are provided as a Source Data file.

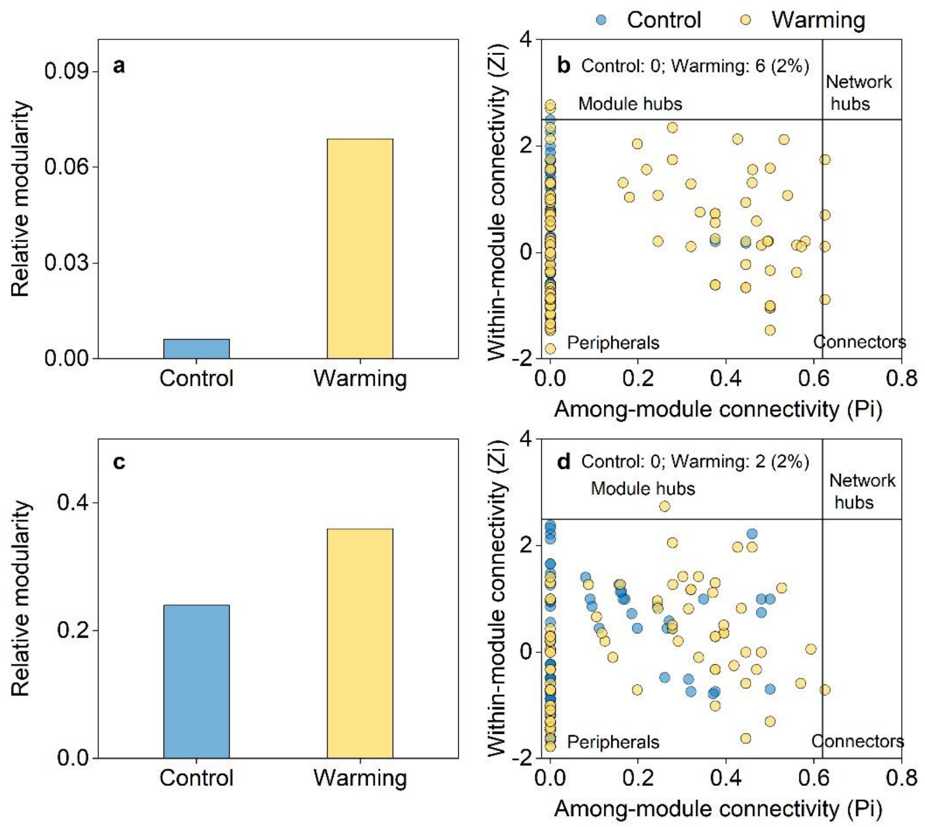

Supplementary Fig. 3 | Relative modularity of networks and keystone node identification. a, c, Relative modularity of prokaryotic (a) and fungal (c) networks in control (blue) and warming (yellow) conditions. **b, d,** Keystone node identification in prokaryotic (b) and fungal (d) networks under the control (blue) and warming (yellow) treatments. Module hubs ($Zi > 2.5$, $Pi \leq 0.62$), connectors ($Zi \leq 2.5$, $Pi > 0.62$) and network hubs ($Zi > 2.5$, $Pi > 0.62$) were referred to as keystone nodes. The total number of keystone nodes was shown in the graph, with its proportion to total network nodes in parentheses. Source data are provided as a Source Data file.

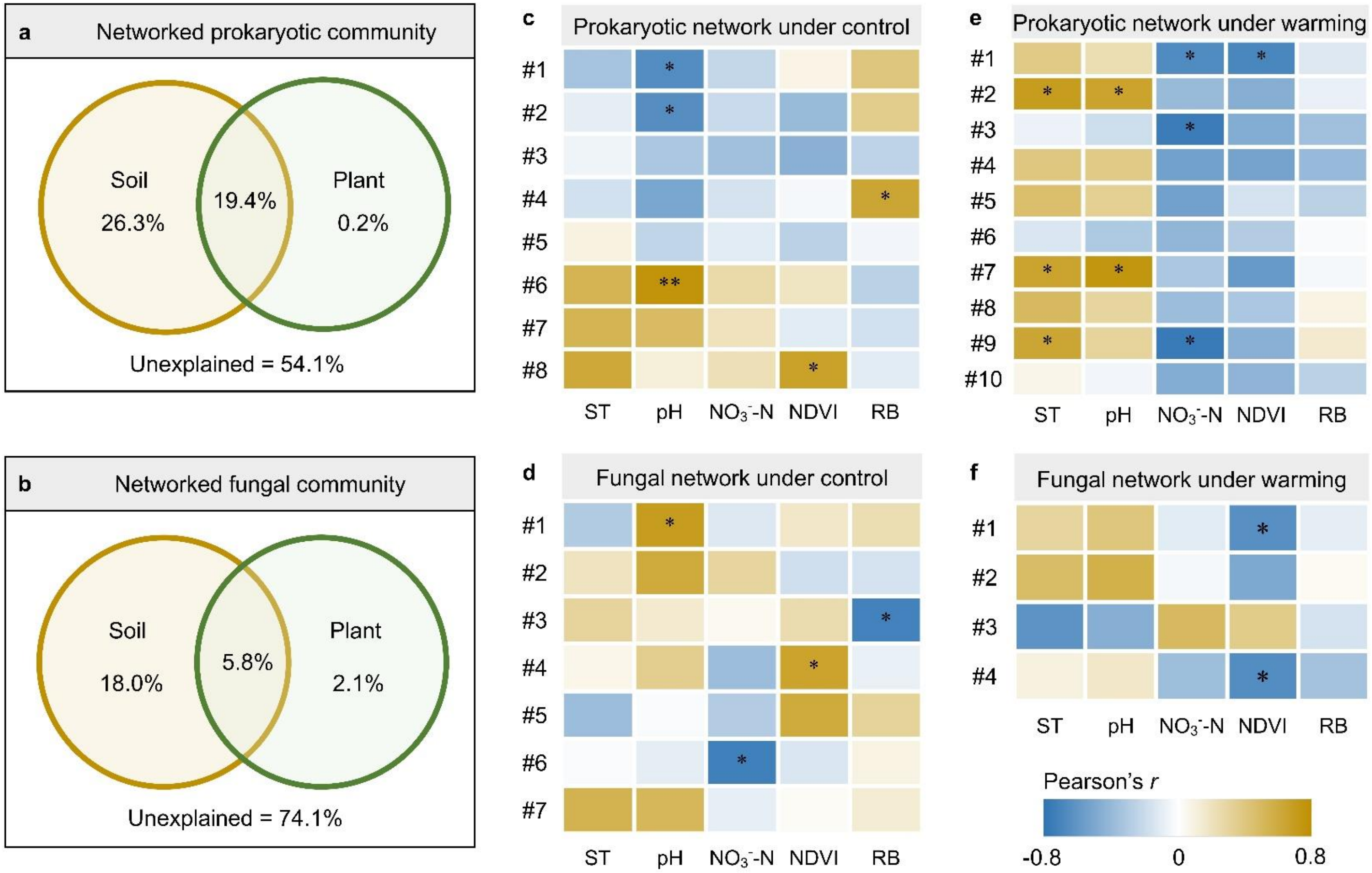

Supplementary Fig. 4 | Connections of networked topsoil microbial communities with environmental factors. a, b, Redundancy analysis-based variation partitioning analysis showing relative contributions of soil and plant variables to the variations of prokaryotic (a) and fungal (b)

communities remained in the networks under the control and warming treatments. Soil variables include soil temperature, soil pH, and NO_3^--N content. Plant variables include vegetation growth reflected by Normalized Difference Vegetation Index (NDVI) and root biomass in 0-10 cm soil. The circles represent the variation explained by the group of soil and plant variables. Numbers in each circle are the unique effects of the corresponding variables, and numbers in the overlapping area of the two circles show the interactions of the two groups. **c-f,** Correlations of module eigengenes with soil and plant variables in the prokaryotic (c, e) and fungal (d, f) networks under control (c, d) and warming (e, f). Rows correspond to eigengenes of major modules in the network (more than five nodes), whereas columns are the environmental factors. Each plot represents the correlation, of which the color indicating its sign and strength. ST, soil temperature; RB, root biomass in 0-10 cm soils. *$P < 0.05$, **$P < 0.01$. Source data are provided as a Source Data file.

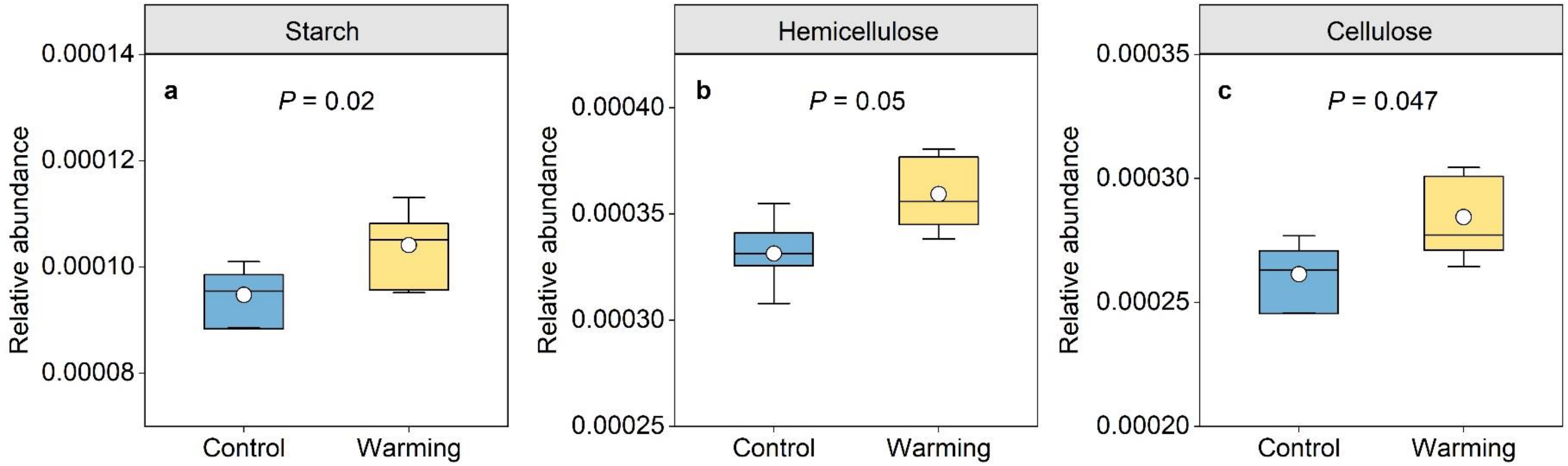

Supplementary Fig. 5 | Effects of experimental warming on topsoil microbial functional genes. a-c, Relative abundance of KEGG Orthology (KO) terms involved in the pathways of starch (a), hemicellulose (b) and cellulose (c) utilization. Involved KOs are determined according to the KEGG database and published literature[9]. Box represents the interquartile range, with blue and yellow indicating control and warming, respectively. Horizontal line and circle within the box show the median and mean value, respectively. The whisker denotes SD (n = 10, independent samples). Paired samples t-tests (two-sided) were used to determine warming effects on means of relative abundance of functional genes. Source data are provided as a Source Data file.

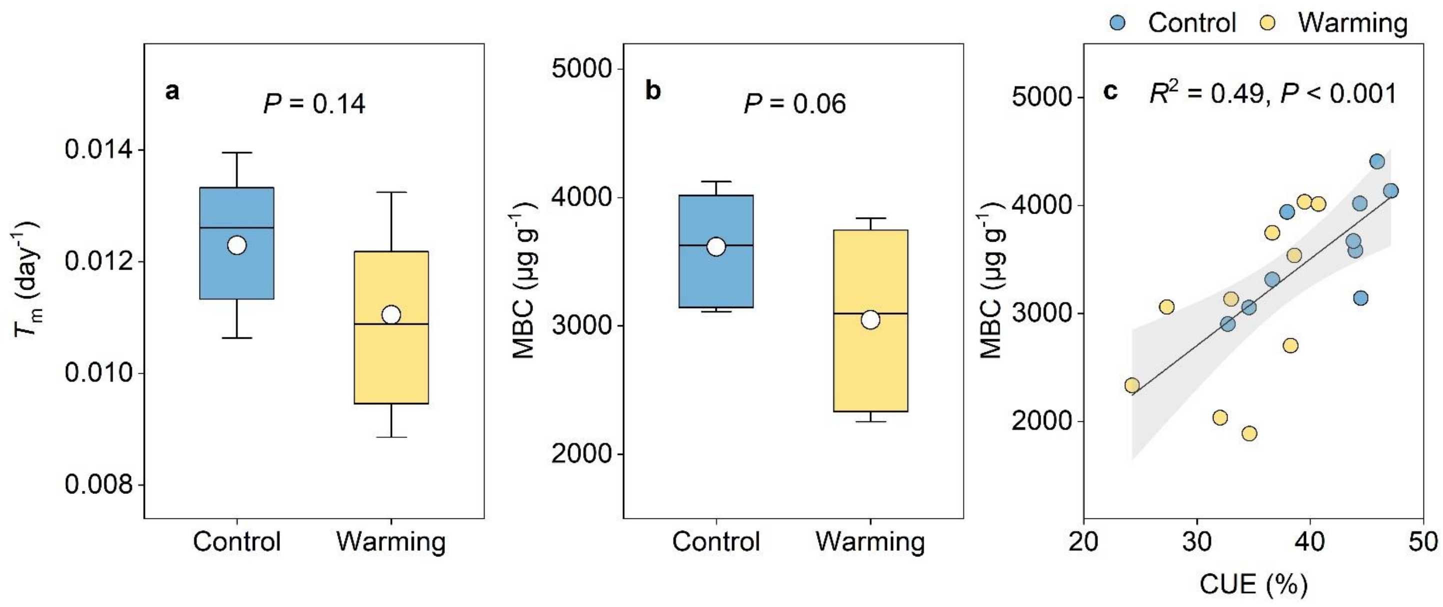

Supplementary Fig. 6 | Responses of topsoil microbial turnover rate (T_m; a) and microbial biomass carbon (MBC; b) to experimental warming, and the relationship between MBC and microbial carbon use efficiency (CUE; c). In panels a and b, box represents the interquartile range, with blue and yellow indicating control and warming, respectively. Horizontal line and circle within the box show the median and mean value, respectively. The whisker denotes SD (n = 10, independent samples). Paired samples t-tests (two-sided) were used to determine the differences in means between the warming and control treatments. In panel c, the solid line represents the regression curve for MBC and CUE, and the grey area shows 95% confidence interval. Source data are provided as a Source Data file.

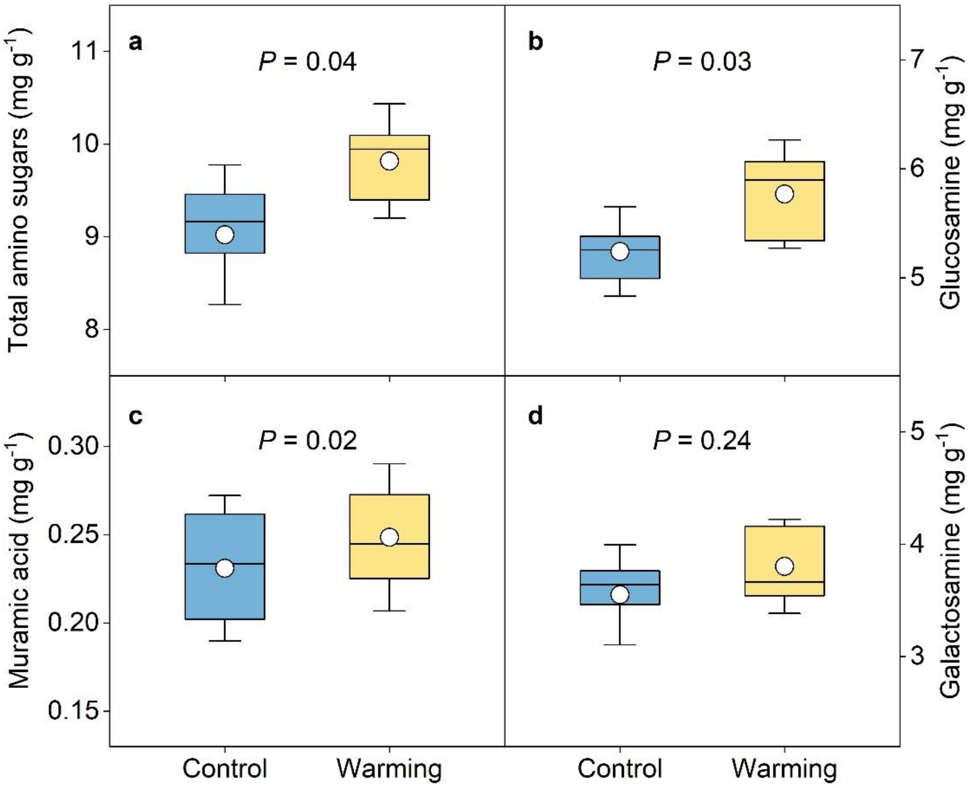

Supplementary Fig. 7 | Effects of experimental warming on contents of topsoil amino sugars. a-d, Contents of total amino sugars (a), glucosamine (b), muramic acid (c) and galactosamine (d). Total amino sugars are the sum of the three individual amino sugar. Box represents the interquartile range, with blue and yellow indicating control and warming, respectively. Horizontal line and circle within the box show the median and mean value, respectively. The whisker denotes SD (*n* = 10, independent samples). Paired samples *t*-tests (two-sided) were used to determine warming effects on means of amino sugar content. Source data are provided as a Source Data file.

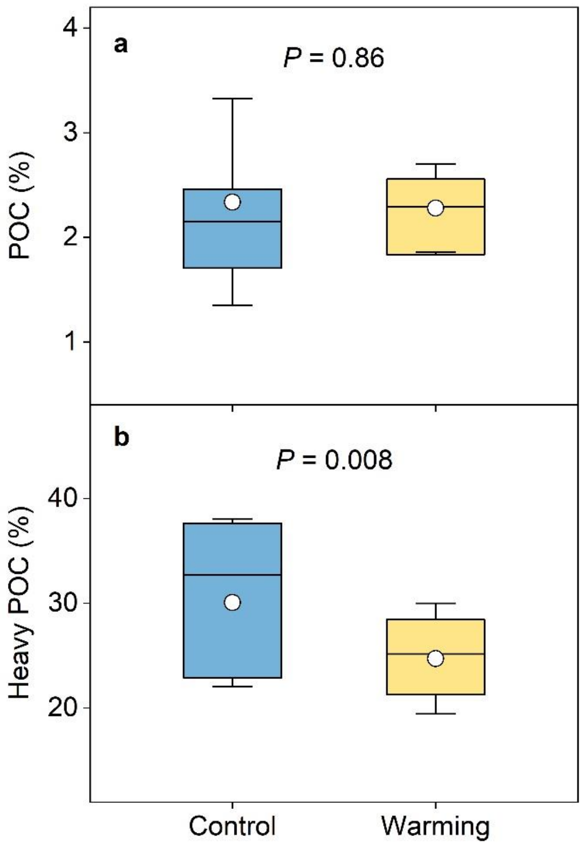

Supplementary Fig. 8 | Warming effects on topsoil carbon distribution in POC and heavy POC. a, Proportion of particulate organic carbon (POC; carbon in the soil fraction lighter than 1.6 g cm^{-3}) to total soil organic carbon. **b,** Proportion of heavy POC (carbon stored in the soil fraction heavier than 1.6 g cm^{-3} and larger than 53 μm) to total soil organic carbon. Box represents the interquartile range, with blue and yellow indicating control and warming, respectively. Horizontal line and circle within the box show the median and mean value, respectively. The whisker denotes SD (n = 10, independent samples). Paired samples t-tests (two-sided) were used to determine warming effects on means of soil carbon distribution. Source data are provided as a Source Data file.

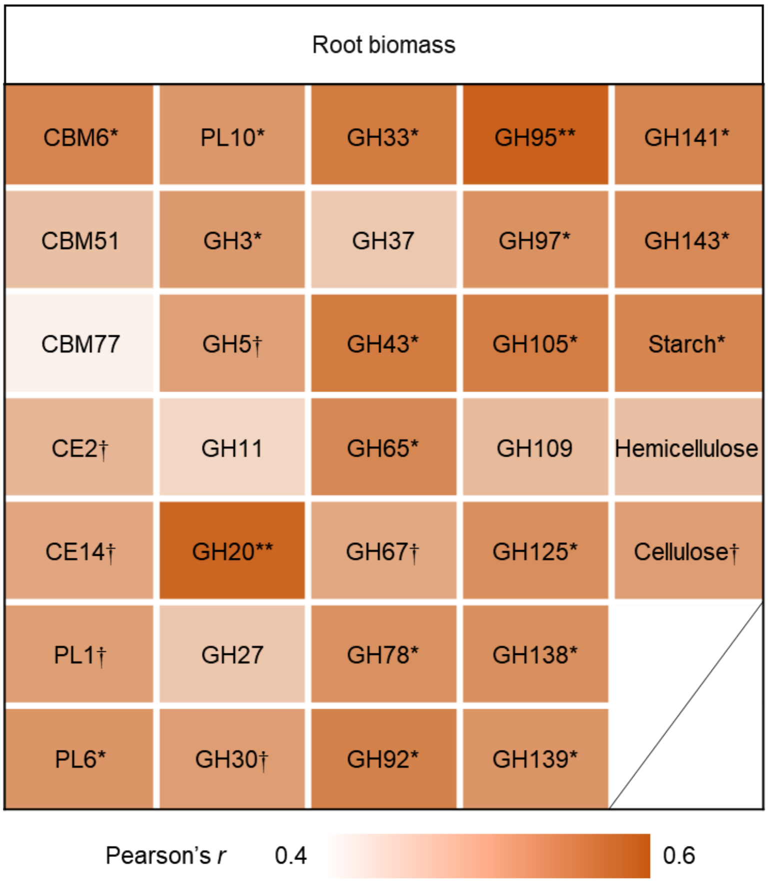

Supplementary Fig. 9 | Pearson's correlations of root biomass with the relative abundance of topsoil microbial functional genes. Root biomass was measured as the live root biomass in 0-10 cm soil. The functional genes were those significantly enriched under the warming treatment as shown in Fig. 2 and Supplementary Fig. 5. CBM, carbohydrate-binding module; CE, carbohydrate esterase; PL, polysaccharide lyase; GH, glycoside hydrolase. $^{\dagger}P < 0.1$, $^{*}P < 0.05$, $^{**}P < 0.01$. Source data are provided as a Source Data file.

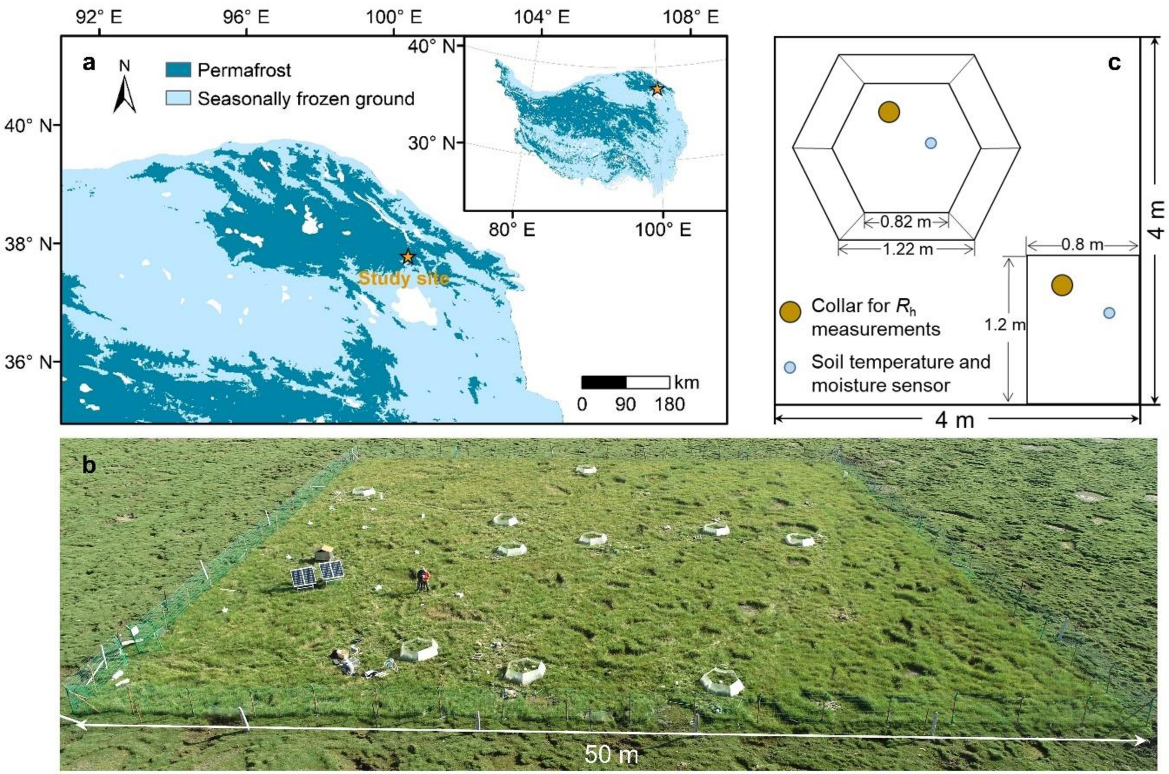

Supplementary Fig. 10 | Location and design of warming experiment in a permafrost ecosystem on the Tibetan Plateau. a, Location of the in situ warming experiment based on the permafrost distribution map on the Tibetan Plateau[10]. The map was drawn using ArcMap 10.6 (Environmental Systems Research Institute, Inc., Redlands, CA, USA) / Zou, D., Zhao, L., Sheng, Y., Chen, J., Hu, G., Wu, T., Wu, J., Xie, C., Wu, X., Pang, Q., Wang, W., Du, E., Li, W., Liu, G., Li, J., Qin, Y., Qiao, Y., Wang, Z., Shi, J., and Cheng, G.: A new map of permafrost distribution

on the Tibetan Plateau, The Cryosphere, 11, 2527–2542, https://doi.org/10.5194/tc-11-2527-2017, 2017 (https://tc.copernicus.org/articles/11/2527/2017/) / CC BY (https://creativecommons.org/licenses/by/3.0/deed.en). **b**, Overview of the experimental site. The warming experiment followed a paired design, with ten 4 × 4 m blocks being randomly established in a 50 × 50 m fenced area. Photo was taken by Bin Wei. **c,** Design of experimental block. Within each block, a 0.8 × 1.2 m plot located at one corner was arranged as control, and an open-top chamber (OTC) at the diagonally opposite corner was arranged as the warming plot. In each plot, a PVC collar (20 cm diameter, 62 cm high) was inserted into the soil to a depth of 60 cm to determine heterotrophic respiration (R_h). Soil temperature and moisture at 5 cm depth were monitored with ECH_2O sensors.

Supplementary Table 1 | Effects of experimental warming on topsoil temperature from 2014 to 2020.

Year	Soil temperature (°C)		*P* value
	Control	Warming	
2014	8.6 ± 0.13	10.8 ± 0.13	1.3e-6
2015	7.7 ± 0.15	9.3 ± 0.20	0.0003
2016	8.4 ± 0.16	9.4 ± 0.26	0.0090
2017	7.5 ± 0.15	8.6 ± 0.27	0.0030
2018	7.7 ± 0.14	8.5 ± 0.25	0.0200
2019	6.5 ± 0.14	7.5 ± 0.22	0.0030
2020	6.2 ± 0.14	7.7 ± 0.16	0.0002

Data are reported as means ± standard errors (n = 10, independent samples). Soil temperature is the mean value in growing season (May to October) measured by ECH_2O sensors at 5 cm depth. Data for 2014-2016 were obtained from ref. 11, and data for 2020 were derived from ref. 12. Paired samples t-tests (two-sided) were conducted to compare means of soil temperature between the warming and control treatments. All P values are < 0.05, indicating significant differences between the two groups.

Supplementary Table 2 | Effects of experimental warming on a series of plant and soil variables.

Variables	Control	Warming	*P* value
NDVI	0.40 ± 0.004	0.42 ± 0.01	**0.04**
Root biomass (g m^{-2})	474.4 ± 30.1	564.1 ± 32.6	**0.01**
Soil moisture (v/v %)	48.2 ± 2.0	47.5 ± 2.0	0.82
SOC (g kg^{-1})	196.9 ± 4.7	193.4 ± 6.0	0.67
DOC (μg g^{-1})	291.4 ± 20.8	326.8 ± 33.0	0.37
NH_4^+-N (mg kg^{-1})	53.9 ± 4.3	56.5 ± 4.5	0.69
NO_3^--N (mg kg^{-1})	16.2 ± 1.3	21.6 ± 1.8	**0.01**
pH	6.07 ± 0.05	6.18 ± 0.07	**0.06**

Data are reported as means ± standard errors (n = 10, independent samples). Root biomass is the value in 0-10 cm soils. Soil moisture is the mean value measured in 2019 (May to October) by ECH_2O sensors at 5 cm depth. NDVI, Normalized Difference Vegetation Index; SOC, soil organic carbon; DOC, dissolved organic carbon. All variables were measured for soil samples collected at 2019. Paired samples *t*-tests (two-sided) were conducted to compare means of plant and soil variables between the warming and control treatments. Bold texts indicate significant ($P < 0.05$) or marginally significant ($P < 0.1$) effects.

Supplementary Table 3 | Significance tests of warming effects on topsoil microbial community composition.

	MRPP		ANOSIM		Adonis	
	A	*P*	*R*	*P*	*F*	*P*
Prokaryotes	-0.003	0.83	-0.03	0.69	0.90	0.84
Fungi	0.001	0.31	-0.001	0.47	1.07	0.29
Networked prokaryotes	0.36	**0.001**	0.95	**0.001**	29.68	**0.001**
Networked fungi	0.13	**0.001**	0.67	**0.001**	6.67	**0.001**

MRPP, multi-response permutation procedure; ANOSIM, analysis of similarities; Adonis, permutational multivariate analysis of variance. The permutation tests were conducted based on Bray–Curtis distance. Networked prokaryotes and fungi represent prokaryotic and fungal taxa retained in the networks. Bold texts indicate significant effects ($P < 0.05$).

Supplementary Table 4 | Topological properties of topsoil prokaryotic and fungal networks under control and warming conditions.

	Network topological characteristics	Prokaryotes		Fungi	
		Control	Warming	Control	Warming
Numbers of ASVs[a]		976	931	170	159
Empirical network	Similarity threshold	0.91	0.91	0.79	0.79
	Total nodes	305	388	132	116
	Total links	213	454	345	376
	R square of power law	0.998	0.993	0.92	0.93
	Average degree (avgK)	1.40	2.34	5.23	6.48
	Average clustering coefficient (avgCC)	0.06	0.13	0.30	0.30
	Average path distance (GD)	2.94	4.97	3.73	3.87
	Density (D)	0.005	0.006	0.04	0.06
	Connectedness (Con)	0.01	0.17	0.33	0.60
	No. of modules	106	102	20	13
	Modularity (M)	0.972	0.77	0.41	0.38
Random networks[b]	avgCC ± SD	0 ± 0	0 ± 0.001	0.002 ± 0.004	0.001 ± 0.004
	GD ± SD	3.79 ± 1.05	4.94 ± 0.13	3.03 ± 0.07	2.82 ± 0.06
	Con ± SD	0.02 ± 0.01	0.61 ± 0.04	0.93 ± 0.04	0.97 ± 0.04
	M ± SD	0.966 ± 0.01	0.72 ± 0.01	0.33 ± 0.01	0.28 ± 0.01

[a]Number of ASVs used for network construction, i.e., ASVs presented in all the 10 samples under control or warming conditions.

[b]100 random networks were generated by rewiring the links of the corresponding empirical network. Mean values and standard derivations (SD) of the 100 random networks were shown.

Supplementary Table 5 | List of the CAZy families involved in the degradation of several plant- and microbial-derived compounds.

CAZy gene family	Substrates	References
AA3	PCW (Lignin)	13,14
CE4	PCW (Hemicellulose);<br>FCW (Chitin); BCW (peptidoglycan)	14,15,16
GH4	PCW (Hemicellulose)	17
GH57	Starch	13
GH77	Starch	18
GH85	FCW (Chitin)	19
GH103	BCW (peptidoglycan)	14
CBM6	Cellulose-binding	20
CBM51	Starch	13
CE2	PCW (Hemicellulose)	14,17
PL1	PCW (Pectin)	15,17
PL10	PCW (Pectin)	17
GH3	PCW (Hemicellulose; Cellulose)	14,17
GH5	PCW (Cellulose)	13,14
GH11	PCW (Hemicellulose)	19,20
GH20	FCW (Chitin)	14,15,17
GH27	PCW (Hemicellulose)	20
GH30	PCW (Hemicellulose)	14
GH37	Starch	13,21
GH43	PCW (Hemicellulose)	13,14,17
GH65	Starch	15
GH67	PCW (Hemicellulose)	14,17
GH78	PCW (Pectin)	15,17
GH95	PCW (Hemicellulose)	14,20
GH97	Starch	13,15
GH105	PCW (Pectin)	15,17
GH125	Starch	13
GH138	PCW (Pectin)	22,23
GH139	PCW (Pectin)	23
GH141	PCW (Hemicellulose)	13
GH143	PCW (Pectin)	22

PCW, plant cell wall; FCW: fungal cell wall; BCW, bacterial cell wall.

Supplementary References

1. Barabási, A.-L. & Oltvai, Z. N. Network biology: understanding the cell's functional organization. *Nat. Rev. Genet.* **5,** 101-113 (2004).
2. Deng, Y. et al. Molecular ecological network analyses. *BMC Bioinformatics* **13,** 113 (2012).
3. Alon, U. Biological networks: the tinkerer as an engineer. *Science* **301,** 1866-1867 (2003).
4. Yuan, M. M. et al. Climate warming enhances microbial network complexity and stability. *Nat. Clim. Chang.* **11,** 343-348 (2021).
5. Olesen, J. M., Bascompte, J., Dupont, Y. L. & Jordano, P. The modularity of pollination networks. *Proc. Natl. Acad. Sci. U. S. A.* **104,** 19891-19896 (2007).
6. Guimerà, R. & Nunes Amaral, L. A. Functional cartography of complex metabolic networks. *Nature* **433,** 895-900 (2005).
7. Shi, S. et al. The interconnected rhizosphere: high network complexity dominates rhizosphere assemblages. *Ecol. Lett.* **19,** 926-936 (2016).
8. Zhou, J. et al. Phylogenetic molecular ecological network of soil microbial communities in response to elevated CO_2. *mBio* **2,** e00122-11 (2011).
9. Kang, L. et al. Metagenomic insights into microbial community structure and metabolism in alpine permafrost on the Tibetan Plateau. *Nat. Commun.* **15,** 5920 (2024).
10. Zou, D. et al. A new map of permafrost distribution on the Tibetan Plateau. *Cryosphere* **11,** 2527-2542 (2017).

11. Li, F. et al. Warming alters surface soil organic matter composition despite unchanged carbon stocks in a Tibetan permafrost ecosystem. *Funct. Ecol.* **34,** 911-922 (2020).

12. Wei, B. et al. Experimental warming altered plant functional traits and their coordination in a permafrost ecosystem. *New Phytol.* **240,** 1802-1816 (2023).

13. Donhauser, J., Qi, W., Bergk-Pinto, B. & Frey, B. High temperatures enhance the microbial genetic potential to recycle C and N from necromass in high-mountain soils. *Glob. Chang. Biol.* **27,** 1365-1386 (2021).

14. López-Mondéjar, R. et al. Metagenomics and stable isotope probing reveal the complementary contribution of fungal and bacterial communities in the recycling of dead biomass in forest soil. *Soil Biol. Biochem.* **148,** 107875 (2020).

15. Dai, Z. et al. Metagenomic insights into soil microbial communities involved in carbon cycling along an elevation climosequences. *Environ. Microbiol.* **23,** 4631-4645 (2021).

16. Pascual, S. & Planas, A. Carbohydrate de-N-acetylases acting on structural polysaccharides and glycoconjugates. *Curr. Opin. Chem. Biol.* **61,** 9-18 (2021).

17. Cardenas, E. et al. Forest harvesting reduces the soil metagenomic potential for biomass decomposition. *ISME J.* **9,** 2465-2476 (2015).

18. Christensen, S. J. et al. Bioinformatics and functional selection of GH77 4-α-glucanotransferases for potato starch modification. *New Biotech.* **79,** 39-49 (2024).

19. Zhao, Z., Liu, H., Wang, C. & Xu, J.-R. Erratum to: comparative analysis of

fungal genomes reveals different plant cell wall degrading capacity in fungi. *BMC Genomics* **15,** 6 (2014).

20. Chang, H.-X., Yendrek, C. R., Caetano-Anolles, G. & Hartman, G. L. Genomic characterization of plant cell wall degrading enzymes and *in silico* analysis of xylanses and polygalacturonases of *Fusarium virguliforme*. *BMC Microbiol.* **16,** 147 (2016).

21. Shrestha, P. et al. Complete genome sequence and comparative genome analysis of *Variovorax* sp. strains PAMC28711, PAMC26660, and PAMC28562 and trehalose metabolic pathways in Antarctica isolates. *Int. J. Microbiol.* **2022,** 5067074 (2022).

22. Zheng, J. et al. dbCAN-seq update: CAZyme gene clusters and substrates in microbiomes. *Nucleic Acids Res.* **51,** D557-D563 (2023).

23. Ndeh, D. et al. Complex pectin metabolism by gut bacteria reveals novel catalytic functions. *Nature* **544,** 65-70 (2017).
