# Supplementary material for: Dual roles of microbes in mediating soil carbon dynamics in response to warming
Source: Nat Commun. 2024 Jul 31;15:6439. doi: 10.1038/s41467-024-50800-4 (PMC11291496; doi:10.1038/s41467-024-50800-4)
Supplement: Supplementary file 3 — Reporting Summary [file 41467_2024_50800_MOESM3_ESM.pdf]

nature portfolio

Corresponding author(s): Yuanhe Yang

Last updated by author(s): Jul 11, 2024

Reporting Summary

Nature Portfolio wishes to improve the reproducibility of the work that we publish. This form provides structure for consistency and transparency in reporting. For further information on Nature Portfolio policies, see our Editorial Policies and the Editorial Policy Checklist.

Statistics

For all statistical analyses, confirm that the following items are present in the figure legend, table legend, main text, or Methods section.

n/a	Confirmed	
☐	☒	The exact sample size (*n*) for each experimental group/condition, given as a discrete number and unit of measurement
☐	☒	A statement on whether measurements were taken from distinct samples or whether the same sample was measured repeatedly
☐	☒	The statistical test(s) used AND whether they are one- or two-sided<br>*Only common tests should be described solely by name; describe more complex techniques in the Methods section.*
☒	☐	A description of all covariates tested
☐	☒	A description of any assumptions or corrections, such as tests of normality and adjustment for multiple comparisons
☐	☒	A full description of the statistical parameters including central tendency (e.g. means) or other basic estimates (e.g. regression coefficient) AND variation (e.g. standard deviation) or associated estimates of uncertainty (e.g. confidence intervals)
☐	☒	For null hypothesis testing, the test statistic (e.g. *F*, *t*, *r*) with confidence intervals, effect sizes, degrees of freedom and *P* value noted<br>*Give P values as exact values whenever suitable.*
☒	☐	For Bayesian analysis, information on the choice of priors and Markov chain Monte Carlo settings
☒	☐	For hierarchical and complex designs, identification of the appropriate level for tests and full reporting of outcomes
☐	☒	Estimates of effect sizes (e.g. Cohen's *d*, Pearson's *r*), indicating how they were calculated

Our web collection on statistics for biologists contains articles on many of the points above.

Software and code

Policy information about availability of computer code

Data collection	No software was used in this study for data collection.
Data analysis	TrimGalore v0.6.10, USEARCH v11, QIIME2 v2024.2, Trimmomatic v0.39, MEGAHIT v1.2.9, prodigal v2.6.3, MMseqs2, run_dbcan and DIAMOND v0.9.14 were used to process the sequencing data; R 4.1.0 was used for statistical analyses.<br>Network construction and analyses were all carried out using the integrated network analysis pipeline (iNAP) at https://inap.denglab.org.cn/.<br>Linear discriminant analysis (LDA) effect size (LEfSe) analyses were carried out at http://galaxy.biobakery.org/.

For manuscripts utilizing custom algorithms or software that are central to the research but not yet described in published literature, software must be made available to editors and reviewers. We strongly encourage code deposition in a community repository (e.g. GitHub). See the Nature Portfolio guidelines for submitting code & software for further information.

Data

Policy information about availability of data

All manuscripts must include a data availability statement. This statement should provide the following information, where applicable:

- Accession codes, unique identifiers, or web links for publicly available datasets
- A description of any restrictions on data availability
- For clinical datasets or third party data, please ensure that the statement adheres to our policy

All data supporting the findings are available in the Figshare data repository (https://doi.org/10.6084/m9.figshare.25974622.v2) and Supplementary Information.

The sequence data generated in this study have been deposited in the NCBI Sequence Read Archive (SRA) under accession number PRJNA1113361. Source data are provided with this paper.

Research involving human participants, their data, or biological material

Policy information about studies with human participants or human data. See also policy information about sex, gender (identity/presentation), and sexual orientation and race, ethnicity and racism.

Reporting on sex and gender	n/a
Reporting on race, ethnicity, or other socially relevant groupings	n/a
Population characteristics	n/a
Recruitment	n/a
Ethics oversight	n/a

Note that full information on the approval of the study protocol must also be provided in the manuscript.

Field-specific reporting

Please select the one below that is the best fit for your research. If you are not sure, read the appropriate sections before making your selection.

☐ Life sciences ☐ Behavioural & social sciences ☒ Ecological, evolutionary & environmental sciences

For a reference copy of the document with all sections, see nature.com/documents/nr-reporting-summary-flat.pdf

Ecological, evolutionary & environmental sciences study design

All studies must disclose on these points even when the disclosure is negative.

Study description	This study provided thorough experimental evidence about microbial responses to experimental warming, and illustrated microbial roles in affecting soil C cycling based on an in situ open-top chamber (OTC) warming experiment. The field warming experiment followed a paired design with two treatments (control and warming) and ten replicates for each treatment.
Research sample	We collected topsoil (0-10 cm) samples from each control and warming plot to examine warming effects on soil microbial attributes. This soil layer was chosen because OTC devices primarily warm surface soils. Until sampling, the soils in warming plots have been continually warmed by OTCs for six years in 2019, and for seven years in 2020.
Sampling strategy	Sample size was 10 for the control treatment and 10 for the warming treatment, which was determined by the replicates of the in situ experimental plot. During soil sampling, three randomized soil cores at 0-10 cm depth were collected from each plot and mixed as a composite. In total, 20 soil samples were acquired (10 from control plots and 10 from warming plots) in each year of 2019 and 2020, which were transferred to the laboratory immediately in a cooler.
Data collection	Field data were collected by Bin Wei, including measurements of heterotrophic respiration using a LI-8100A automated soil CO2 flux system (Li-Cor Inc., Lincoln, NE, USA), and Normalized Difference Vegetation Index using a multi-spectral camera (ADC Lite, Tetracam, Chatsworth, CA, USA). In laboratory, measurements of soil physicochemical properties, root biomass and microbial physiology were performed by Shuqi Qin, Dianye Zhang and Bin Wei following standard protocols. Amplicon sequencing and metagenomic sequencing were conducted at Guangdong Magigene Biotechnology Co., Ltd. (Guangzhou, China).
Timing and spatial scale	Heterotrophic respiration was measured between 9:00 a.m. and 12:00 p.m. (local time) twice to three times per month from May to October in 2020. Soil sampling was conducted in July of 2019 and 2020. After sampling in 2019, amplicon sequencing as well as measurements of soil physicochemical properties were performed. After sampling in 2020, metagenomic sequencing and measurements of microbial physiology properties were conducted.
Data exclusions	No data were excluded from the analyses.
Reproducibility	Heterotrophic respiration was measured twice to three times per month from May to October in 2020. For each measurement, the respiration rate was higher under the warming treatment. The study design, experimental methods, and statistics are described in detail, and all data for reproducing the results are available publicly.
Randomization	For the design of in situ warming experiment, 10 blocks were randomly established in a 50 × 50 m fenced area. During soil sampling, three randomized soil cores at 0-10 cm depth were collected from each plot.
Blinding	Not relevant because data collection and analysis were conducted using standardized protocols, minimizing the potential for observer bias or influence on the conclusions.

Did the study involve field work? ☒ Yes ☐ No

Field work, collection and transport

Field conditions	The study site is cold with mean annual air temperature of -3.4°C. Mean annual precipitation amounts to 466 mm and mainly occurs during growing season.
Location	The study site is located at the foot of Wayan Mountain (37°45′N, 100°05′E; 3800 m above sea level) in Gangca County, Qinghai Province, China.
Access & import/export	The study site can be easily reached by cars, and the soil samples collected were transferred to the laboratory immediately in a cooler.
Disturbance	We minimized the disturbance to soil profile by using small size of soil corer during sampling.

Reporting for specific materials, systems and methods

We require information from authors about some types of materials, experimental systems and methods used in many studies. Here, indicate whether each material, system or method listed is relevant to your study. If you are not sure if a list item applies to your research, read the appropriate section before selecting a response.

Materials & experimental systems

n/a	Involved in the study	
☒	☐	Antibodies
☒	☐	Eukaryotic cell lines
☒	☐	Palaeontology and archaeology
☒	☐	Animals and other organisms
☒	☐	Clinical data
☒	☐	Dual use research of concern
☒	☐	Plants

Methods

n/a	Involved in the study	
☒	☐	ChIP-seq
☒	☐	Flow cytometry
☒	☐	MRI-based neuroimaging

Plants

Seed stocks	n/a
Novel plant genotypes	n/a
Authentication	n/a
